# Gestational Changes in Placental Iron Homeostasis are Associated With Ferroptosis‐Associated Redox Signaling and Trophoblast Behavior

**DOI:** 10.1096/fj.202504881RR

**Published:** 2026-09-28

**Authors:** Wenxin Jiang, Yike Yang, Xiao Yuan, Wei Deng, Mark D. Kilby, Philip N. Baker, Chao Tong, Yi Yang

**Affiliations:** ^1^ Growth, Development and Mental Health Center of Children and Adolescents, National Clinical Research Center for Child Health and Disorders, Ministry of Education Key Laboratory of Child Development and Disorders Children's Hospital of Chongqing Medical University Chongqing China; ^2^ Department of Obstetrics Peking University Third Hospital Beijing China; ^3^ Fetal Medicine Centre Birmingham Women's and Children's Foundation Trust Birmingham UK; ^4^ College of Medical and Health Sciences University of Birmingham Birmingham UK; ^5^ Faculty of Medicine and Health Sciences University of East Anglia, Norwich Research Park Norwich UK

**Keywords:** ferroptosis‐associated redox signaling, iron metabolism, lipid peroxidation, placental iron homeostasis, placentation, trophoblast invasion

## Abstract

Well‐regulated trophoblast proliferation, migration, invasion, and cell turnover are essential for normal placental development in humans and rodents. Given the established role of oxidative stress in placentation and the redox activity of iron, we investigated whether ferroptosis‐associated redox signaling contributes to trophoblast function and placental development. Placental iron profiling revealed significantly elevated total and ferrous iron levels in first‐trimester human villi compared with term placentas, accompanied by the transcript levels of several iron uptake‐ and reduction‐related genes, including *TFRC*, *DMT1*, *ZIP8*, *STEAP3*, and *STEAP4*, which were elevated in first‐trimester villi. Murine placentas also exhibited gestational changes in iron abundance and iron‐homeostasis‐related gene expression. HO‐1 protein abundance was highest during early gestation and declined thereafter, suggesting a potential association between heme degradation and gestational iron homeostasis. In contrast, placental labile iron pool (LIP) levels showed only modest, statistically nonsignificant changes across gestation, suggesting a relatively stable redox‐active iron pool. Immunofluorescence analyses demonstrated spatially distinct expression of ferroptosis‐associated regulators, with ACSL4 enriched in invasive trophoblast populations and GPX4 predominantly localized in surrounding decidual tissues, suggesting regional heterogeneity in ferroptosis‐associated molecular features during placentation. Functional studies in HTR‐8/SVneo trophoblast cells further demonstrated that mild ferroptosis‐associated redox perturbation induced by low‐dose erastin or ferrous iron enhanced trophoblast migration and invasion without overt cytotoxicity. These effects were attenuated by ferrostatin‐1, deferoxamine mesylate (DFOM), or the mitochondria‐targeted antioxidant MitoQ and were blunted following *FTH1* and *TFRC* knockdown, indicating that trophoblast responsiveness depends on iron availability and ferroptosis‐associated redox signaling. Importantly, these pro‐invasive effects were preserved under physiologically relevant hypoxic conditions. Together, our findings support a model in which gestational changes in placental iron homeostasis are associated with ferroptosis‐related molecular features, while experimentally induced iron‐dependent redox signaling modulates trophoblast behavior. Rather than inducing overt ferroptotic cell death, sublethal iron‐dependent redox perturbation may act as a signaling mechanism influencing trophoblast function during placental development.

## Graphical abstract

This graphical abstract summarizes gestational changes in placental iron homeostasis and their relationship to ferroptosis‐associated molecular features. In trophoblast cells, experimentally induced sublethal iron‐dependent redox perturbation modulates migration and invasion without causing overt ferroptotic cell death, whereas iron chelation or reduced TFRC expression attenuates these cellular behaviors. These findings suggest that controlled iron‐dependent redox signaling may influence trophoblast function during placental development.

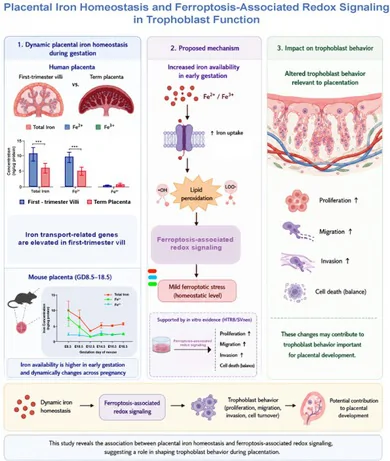

## 1 Introduction

The placenta is a transient, multifunctional organ unique to pregnancy that serves as the interface between the mother and fetus. Its core architecture comprises trophoblast cells embedded within a scaffold of mesenchymal cells and fetal vasculature at the maternal–fetal interface [1]. Humans, rats, and mice all possess a hemochorial placental type, characterized by extensive trophoblast invasion and spiral artery remodeling [2]. Impairment of this developmental process, particularly defective trophoblast invasion and incomplete spiral artery remodeling during early pregnancy, is strongly associated with major obstetric complications, including fetal growth restriction (FGR) and preeclampsia (PE) [3, 4].

Oxidative stress (OS) is a well‐established regulator of placentation [5, 6, 7, 8, 9]. Previous work from our group demonstrated that mild oxidative stress supports trophoblast function during early gestation [10], and we further reported that 4‐hydroxynonenal (4‐HNE), a marker of lipid peroxidation and oxidative stress, peaks in first‐trimester villi and gradually declines thereafter [11]. Despite these findings, the mechanisms regulating placental lipid peroxidation and oxidative stress remain incompletely understood. Given its central role in redox reactions, iron represents a plausible regulator of these processes. Iron exists in two oxidative states, ferrous (Fe^2+^) and ferric (Fe^3+^). In its free ferrous form, iron is highly redox active and promotes reactive oxygen species (ROS) production and lipid peroxidation through the Fenton reaction [12, 13].

During pregnancy, maternal iron demand increases substantially to support placental and fetal development as well as maternal erythropoiesis [14]. Approximately 90 mg of iron is retained within the placenta, whereas about 270 mg is transferred to the fetus [15, 16]. To meet this demand, maternal transferrin levels progressively increase during gestation, facilitating iron delivery to the placenta. Placental iron transport begins with uptake by transferrin receptor (TFRC) on syncytiotrophoblasts, followed by endocytosis, acidification‐dependent iron release, and reduction to Fe^2+^ by ferrireductases such as STEAP3 and STEAP4. Iron is subsequently transported across the cytoplasm via divalent metal transporter 1 (DMT1) or Zrt/Irt‐like proteins (ZIP8/14) and exported by ferroportin (FPN) for transfer to the fetal circulation [15, 17, 18, 19]. Within cells, excess iron is primarily stored in ferritin to limit participation in radical‐generating reactions, whereas the labile iron pool (LIP), composed of redox‐active intracellular iron, is closely linked to iron‐dependent ROS production and lipid peroxidation.

Ferroptosis is an iron‐dependent form of regulated cell death characterized by excessive lipid peroxide accumulation and oxidative membrane damage. Acyl‐CoA synthetase long‐chain family member 4 (ACSL4) facilitates ferroptosis by promoting incorporation of polyunsaturated fatty acids (PUFAs) into membrane phospholipids [20], whereas glutathione peroxidase 4 (GPX4) suppresses ferroptosis by detoxifying lipid hydroperoxides [21]. Emerging evidence suggests that ferroptosis‐associated signaling may exert context‐dependent physiological functions beyond overt cell death. Given the gestational changes in placental iron homeostasis and ferroptosis‐associated molecular features, we hypothesized that ferroptosis‐associated redox signaling may participate in placentation and regulate trophoblast behavior during early pregnancy.

## 2 Methods

### 2.1 Human Placental Tissue Collection

First‐trimester placental villi were obtained from pregnant women undergoing elective surgical termination for non‐medical reasons at the Department of Obstetrics, The First Affiliated Hospital of Chongqing Medical University. Term placentas were collected immediately following cesarean section delivery. All tissue specimens were promptly rinsed with cold 0.9% sterile normal saline (NS), subsequently flash‐frozen in liquid nitrogen, and stored at −80°C for further analysis or fixed in 4% formaldehyde for histological processing. This study was approved by the Ethics Committee of The First Affiliated Hospital of Chongqing Medical University (#2018‐113) and was conducted in accordance with the principles of the Declaration of Helsinki. Written informed consent was obtained from all participating subjects.

### 2.2 Animal Experiments

Institute of Cancer Research (ICR) mice were purchased from Huafukang Bioscience Co. Inc. (Beijing, China). Animals were housed under specific pathogen‐free conditions at a constant temperature of 25°C with a 12‐h light/12‐h dark cycle, and provided with standard rodent chow and water ad libitum. Body weight and general health status were monitored daily. To establish timed pregnancies, 8–10‐week‐old ICR female mice were mated with 8–12‐week‐old male mice; the presence of a vaginal plug was designated as gestational day (GD) 0.5. Pregnant mice were euthanized via CO_2_ asphyxiation prior to tissue collection. All animal procedures were performed in compliance with the institutional guidelines of Chongqing Medical University and were approved by the Ethics Committee of The First Affiliated Hospital of Chongqing Medical University.

### 2.3 Immunofluorescence (IF) Staining

Human villi, term placental tissues, and murine fetoplacental units were fixed in 4% paraformaldehyde, dehydrated, and embedded in paraffin. For IF staining, 3‐μm‐thick tissue sections were subjected to antigen retrieval using EDTA buffer (pH 9.0) and incubated with 3% H_2_O_2_ to quench endogenous peroxidase activity. Sections were then incubated with primary antibodies against ferritin (1:200, #ab75973, Abcam, UK), cytokeratin 7 (CK7, 1:100; Servicebio, China), ACSL4 (1:200, #ab155282, Abcam), or GPX4 (1:200, #ab125066, Abcam) overnight at 4°C. After washing, sections were incubated with appropriate fluorescently conjugated secondary antibodies. Nuclei were counterstained with DAPI (Vector Laboratories, USA). Signal was developed using diaminobenzidine (DAB, ZSGB‐BIO, China), and images were captured using a Life EVOS imaging system.

### 2.4 Western Blotting

Tissue samples were homogenized in RIPA lysis buffer (Beyotime Biotechnology, China) supplemented with 1:100 PMSF (Beyotime Biotechnology) on ice, followed by centrifugation at 12 000 × *g* for 15 min at 4°C. The protein concentration of the supernatant was quantified, normalized, and mixed with Laemmli sample buffer (LDS, #4006028, Bio‐Rad, USA) and dithiothreitol (DTT). Proteins were separated by 10% SDS‐PAGE and transferred onto polyvinylidene difluoride (PVDF) membranes (Merck Millipore, Germany). Membranes were blocked with 5% non‐fat milk in Tris‐buffered saline containing 0.05% Tween‐20 (TBST) for 1 h at room temperature and then probed overnight at 4°C with rabbit monoclonal antibodies against ACSL4 (1:2000, #ab155282, Abcam), rabbit monoclonal antibodies against GPX4 (1:1000, #ab125066, Abcam), or mouse monoclonal antibodies against GAPDH (1:5000, #ab8245, Abcam). After incubation with horseradish peroxidase (HRP)‐conjugated secondary antibodies (goat anti‐mouse or goat anti‐rabbit IgG) for 1 h at room temperature, protein bands were visualized. Band intensity was quantified using the Quantity One System Image Analyzer (Bio‐Rad).

### 2.5 Reverse Transcription and Quantitative Real‐Time PCR (qRT‐PCR)

Total RNA was extracted from cultured cells and placental tissues using TRIzol reagent (Invitrogen, USA) according to the manufacturer's instructions. For cDNA synthesis, 1 μg of total RNA was reverse‐transcribed using oligo‐dT primers (#07912455001, Roche, Germany) and Thermoscript reverse transcriptase (Bio‐Rad). qPCR was performed using SYBR Green master mix (#06924204001, Roche) on an Applied Biosystems PCR system. The primer sequences (Takara, Japan) used for genes including *DMT1, TFRC, ZIP8, ZIP14, STEAP3, STEAP4*, and *FPN* are listed in Table 1, all primers were designed to detect total transcript abundance rather than individual isoforms. The thermal cycling conditions were: 95°C for 10 min, followed by 40 cycles of 95°C for 10 s, 63.3°C for 30 s, and 72°C for 10 s. β‐Actin was used as an internal control. All reactions were performed in triplicate. The threshold cycle (*C*_t_) value was defined as the cycle number at which the fluorescence signal exceeded a predetermined threshold.

**TABLE 1:** Primers used for RT‐qPCR amplification.

	Homo	Mus
	Forward 5′–3′/Reverse 5′–3′	Forward 5′–3′/Reverse 5′–3′
*IREB1/Ireb1*	ACATCGTGCTCACCATTACCAA/TGTTTGCACCAGGTACGATAC	TGTGGGTGGTATTGAAGCAGAA/TTGGTAATGGTGAGCACGATGT
*IREB2/Ireb2*	GGATACGCCTTTGAGTACCTTATT/GGGATCACTGCCACATTCTT	GATTGTGACCGTGCCAATTATC/CTAAAGGCTCTGTCTGGAAGTC
*DMT1/Dmt1*	CCACCATGACAGGAACCTATTC/CACAGCAGCCACCACATATAA	CAGGATGTGGAGCACCTAAC/GAGGATGGGTATGAGAGCAAAG
*ZIP8/Zip8*	GTCATCTGTCCAGCAGTCTTAC/CCAGGCAATCGTCCCTATTT	GGGACTAGCTTTCGGCATTT/CTCTCAGCATGTCGTTCATCTC
*ZIP14/Zip14*	CCTGCTTGGCTTATGGAGAA/CACTGCAGACTTGGAGACATAA	GACCCTTTCCACTGATGATTCT/CCACATCTCTCAGTGCTTCTT
*STEAP3/Steap3*	CATCTTTGTGGCTGTGTTCCG/GCTCTTGCTCTGTAGGGTTGCT	AGAAGTCTGGAGGATCCAGAT/CTCCTTCCAGTTGAGGGAATTAG
*STEAP4/Steap4*	ACCGTTACCCAGGCAATACTCA/TGCTAACAGATGGCAAAGAAGTGA	GGTATTCTCGCTGCCATTCT/TCCCTTGCTATTCTGGTCTTTC
*TFRC/Tfrc*	AGAGGTCGCTGGTCAGTTCG/GCCCATTTCCTTTATGTCTGC	CGTGGAGACTACTTCCGTGCTAC/TGGAGATACATAGGGCGACAGG
*HEPCIDIN/Hepcidin*	CACAACAGACGGGACAACTT/GGGCAGCAGGAATAAATAAGGA	CCAATGCAGAAGAGAAGGAAGA/GATGTGGCTCTAGGCTATGTTT
*FPN/Fpn*	GAGTGGTTCCATCCTCAGTATTT/AGAGCTTGTTTCCCAGAGTATTT	TGATGGGAGCATCAGCAATAA/CTGGGCTAGTCCTGAGAATAGA
*Actin/Actin*	AGATTACTGCTCTGGCTCCTAGC/ACTCATCGTACTCCTGCTTGCT	AGATTACTGCTCTGGCTCCTAGC/ACTCATCGTACTCCTGCTTGCT
*Hmox1*		ACAGAGGAACACAAAGACCAG/GTGTCTGGGATGAGCTAGTG
*Nqo1*		TGAAGAAGAGAGGATGGGAGG/GATGACTCGGAAGGATACTGAAAG
*Fth1*		TCAACCGCCAGATCAACC/AGTTCTTCAGAGCCACATCATCTCGG

### 2.6 Iron Assay

Iron concentration was measured using a commercial Iron Assay Kit (MAK025; Sigma‐Aldrich) according to the manufacturer's protocol. Briefly, a standard curve was generated using 0–10 nmol/well of a 1‐mM iron standard solution. Villi and placental tissues were homogenized in 5 volumes of iron assay buffer and centrifuged at 16 000 × *g* for 10 min at 4°C to remove insoluble debris. For measurement of ferrous iron (Fe^2+^) or total iron, 10 μL of sample or standard was added to a 96‐well plate and the volume was adjusted to 100 μL/well with assay buffer. Then, 5 μL of iron assay buffer (for Fe^2+^) or iron reducer (for total iron) was added to each well and incubated for 30 min at room temperature. Subsequently, 100 μL of iron probe was added to each well and incubated for an additional 60 min at room temperature. The absorbance was measured at 593 nm.

### 2.7 Cell Culture

The immortalized human extravillous trophoblast cell line HTR‐8/SVneo was purchased from the American Type Culture Collection (ATCC, Manassas, VA, USA). Cells were cultured in Roswell Park Memorial Institute (RPMI) 1640 medium (#11875093, Gibco, USA) supplemented with 10% fetal bovine serum (FBS, #ST30‐2602, PAN, Germany) and maintained at 37°C in a humidified atmosphere of 5% CO_2_. Erastin (#s7242, Selleck, USA) and mitoquinone mesylate (MitoQ, #HY‐100116A, MCE, USA) were dissolved in dimethyl sulfoxide (DMSO, Sigma, USA). Iron (II) chloride tetrahydrate (FeCl_2_·4H_2_O, #220299, Sigma) and deferoxamine mesylate (DFOM, #HY‐B0988, MCE) were dissolved in ddH_2_O.

### 2.8 Measurement of Reactive Oxygen Species (ROS)

Intracellular ROS levels were assessed using the fluorescent probe 2′,7′‐dichlorodihydrofluorescein diacetate (DCFH‐DA, #S0033S, Beyotime Biotechnology) as previously described [10]. Briefly, cells were seeded into 96‐well plates at a density of 5000 cells/well. After adherence, cells were treated with the indicated compounds for 24 h. Subsequently, cells were incubated with DCFH‐DA (diluted 1:1000 in RPMI 1640) for 20 min at 37°C and washed twice with phosphate‐buffered saline (PBS). Fluorescence intensity was measured using a fluorescence microplate reader (Thermo Fisher, USA). To account for potential differences in cell number, a parallel CCK‐8 assay was performed to normalize the ROS data.

### 2.9 CCK‐8 Assay

HTR‐8/SVneo cells were seeded into 96‐well plates at 5000 cells/well. After cell attachment, they were treated with the specified compounds for 24 h. The culture medium was then replaced with 100 μL of fresh basal medium containing 10% CCK‐8 reagent (Dojindo, Japan) per well, followed by incubation for 4 h at 37°C. The absorbance was measured at 450 nm using a microplate reader (Thermo Fisher, USA).

### 2.10 EdU Staining

Cell proliferation was assessed using the BeyoClick EdU Cell Proliferation Kit (Beyotime Biotechnology). HTR‐8/SVneo cells were seeded into 96‐well plates (8000 cells/well) and treated with compounds after adherence. Following 20 h of treatment, 20 μM EdU was added to the culture medium for an additional 4 h. Cells were then fixed with 4% paraformaldehyde, permeabilized with 0.3% Triton X‐100, and incubated with Click Reaction Cocktail for 30 min in the dark. Cell nuclei were stained with Hoechst 33342. Images were acquired using fluorescence microscopy, and the number of EdU‐positive cells was quantified using ImageJ software (v1.50i).

### 2.11 Cell Viability Assay

HTR‐8/SVneo cells were seeded into 24‐well plates (10 000 cells/well) and treated as indicated. Cell viability was assessed using a Live/Dead Cell Imaging Kit (Invitrogen) according to the manufacturer's instructions. Briefly, two fluorescent probes from the kit were added to the culture medium (1 drop per 500 μL) and incubated for 15 min at 37°C. Cells were then imaged under a fluorescence microscope. Five random fields of view were captured per group. The numbers of total and dead cells were counted using ImageJ software (v1.50i).

### 2.12 Flow Cytometry

Apoptosis was assessed by flow cytometry as previously described [22]. HTR‐8/SVneo cells (50 000 cells/well) were harvested after treatment by centrifugation at 1000 rpm for 5 min. The cell pellet was resuspended in 500 μL of 1× binding buffer and stained with Annexin V and propidium iodide (PI) using a standard apoptosis detection kit. Stained cells were analyzed immediately using a FACScan flow cytometer (CytoFLEX, China).

### 2.13 Matrigel Transwell Invasion Assay

Cell invasion was evaluated using 24‐well Transwell chambers (8‐μm pore size, BD Falcon) precoated with Matrigel (BD Biosciences). HTR‐8/SVneo cells were resuspended in serum‐free RPMI‐1640 medium containing the respective treatments and seeded into the upper chamber (50 000 cells/well). The lower chamber was filled with complete medium containing 10% FBS as a chemoattractant. After 24 h of incubation, noninvading cells on the upper surface of the membrane were removed with a cotton swab. Cells that had invaded through the Matrigel and migrated to the lower surface were fixed with 4% paraformaldehyde, stained with 0.1% crystal violet, and imaged under a microscope. The number of invaded cells was counted in five random 20× fields per well. Each experiment was conducted.

### 2.14 Cell Migration Assay

HTR‐8/SVneo cells were seeded into 12‐well plates and grown to > 90% confluence. A sterile 200‐μL pipette tip was used to create a straight scratch (“wound”) across the cell monolayer. After washing with PBS to remove detached cells, the cells were treated with the indicated compounds in serum‐free medium. Images of the wound were captured at 0 h and 24 h after scratching using a microscope. The migration area was quantified by measuring the change in wound width using ImageJ software. Each experiment was performed in triplicate.

### 2.15 Labile Iron Pool (LIP) Quantification

Mouse placentas at E12.5, E14.5, and E18.5 were collected and washed three times in ice‐cold PBS to remove residual blood. Placental tissues were then minced into small fragments (< 1 mm^3^) and digested in 0.5 mL TrypLE (one placenta per tube) at 37°C. Following centrifugation at 400 × *g* for 5 min to remove TrypLE, tissues were further digested in collagenase/DNase I solution (1 mg/mL collagenase supplemented with DNase I) at 37°C to obtain single‐cell suspensions. Digestion was terminated by adding DMEM containing 2% FBS.

The resulting cell suspension was filtered through a 70 μm cell strainer and centrifuged at 400 × *g* for 5 min. Cells were first stained with Zombie NIR viability dye (BioLegend) for 15 min at room temperature to distinguish live and dead cells. After washing once with 2% FBS in PBS, cells were incubated with anti‐CD45‐APC and anti‐Ter119‐PE antibodies at room temperature in the dark to exclude hematopoietic cells. Cells were then washed with PBS containing 2% FBS and stained with 0.4 μM Calcein‐AM at 37°C for 30 min.

To quantify the labile iron pool (LIP), each sample was divided into two aliquots following Calcein‐AM staining. One aliquot was treated with 100 μM deferoxamine (DFO) at 37°C for 10 min to chelate intracellular labile iron and recover Calcein fluorescence. Samples were subsequently analyzed by flow cytometry. LIP levels were quantified based on the DFO‐induced recovery of Calcein‐AM fluorescence intensity.

### 2.16 Statistical Analysis

All statistical analyses were performed using GraphPad Prism software (version 7). Data are presented as the mean ± standard error of the mean (SEM). Comparisons between two groups were analyzed using an unpaired Student's *t*‐test. Comparisons among multiple groups were analyzed by one‐way analysis of variance (ANOVA) followed by appropriate post hoc tests as indicated in the figure legends. A *p*‐value of less than 0.05 was considered statistically significant.

## 3 Results

### 3.1 Differences in Iron Homeostasis Between First‐Trimester Human Villi and Term Placentas

We first assessed iron status in human placental tissues from different gestational ages. First‐trimester villi exhibited significantly higher levels of total iron, with a pronounced accumulation of ferrous iron (Fe^2+^), compared to term placentas (Figure 1A). We next examined the transcription of genes involved in different aspects of iron homeostasis, including iron uptake and transport‐related genes (*TFRC*, *DMT1/SLC11A2*, and *ZIP8/SLC39A8*), the iron exporter gene *FPN*, ferrireductases (*STEAP3* and *STEAP4*), and iron regulatory genes (*IREB1/ACO1* and *IREB2*). Compared with term placentas, first‐trimester villi exhibited higher mRNA levels of the iron uptake and transport‐related genes *TFRC*, *DMT1*, and *ZIP8*, as well as the ferrireductases *STEAP3*, and *STEAP4*. The mRNA levels of *IREB1* and *IREB2*, which encode iron regulatory proteins, were also increased in first‐trimester villi (Figure 1B). At the protein level, neither IRP1 nor IRP2 differed significantly between first‐trimester villi and term placentas, indicating that differences observed at the transcript level were not accompanied by detectable changes in total protein abundance (Figure 1C,D). Consistent with the higher iron levels observed in first‐trimester villi, ferritin protein abundance was also increased compared with term placentas (Figure 1E).

**FIGURE 1:**
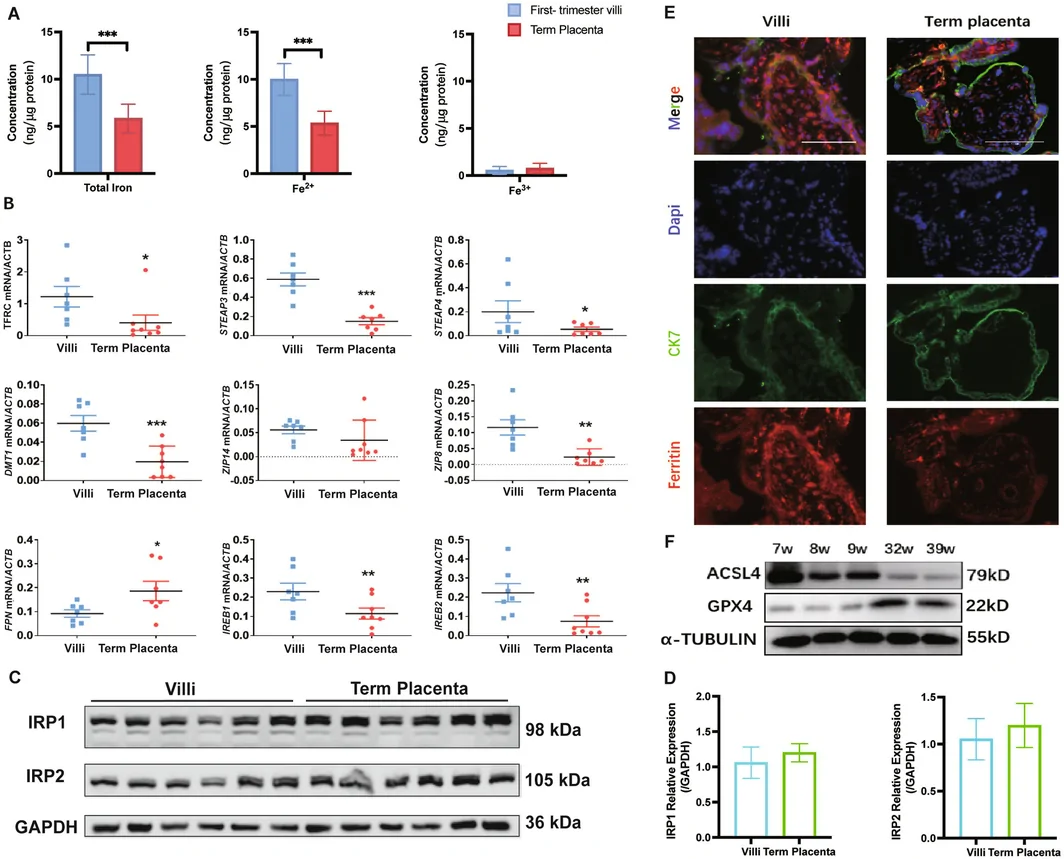
Enhanced iron availability and ferroptosis‐associated signaling in early human placental villi. (A) Iron concentration measurements (total iron, ferrous, and ferric iron) in villi and term placentas (*n* = 8). (B) The transcription levels of iron uptake and transport‐related genes (*TFRC, DMT1, ZIP8, ZIP14*), the iron exporter gene (*FPN*), iron reduction‐related genes (*STEAP3/4*), and iron regulatory genes (*IREB1* and *IREB2*) in first‐trimester villi and term placentas (*n* = 8) were analyzed by qPCR. (C) Immunofluorescence staining of ferritin (iron storage, red), CK7 (trophoblast marker, green), and DAPI (nuclei, blue) in human chorionic villi and term placenta. Scale bar = 100 μm. (D) Western blot analysis of ACSL4 and GPX4 protein levels in different gestational stages. (E) Western blot of IRP1 and IRP2 protein levels in human villi and term placenta. (F) Quantification of IRP1/IRP2 protein levels normalized to GAPDH. Data are presented as mean ± SD (*n* ≥ 3 independent experiments). The *t*‐test, *p < 0.05, **p < 0.01, ***p < 0.001.

Furthermore, ACSL4 protein abundance was significantly higher in first‐trimester villi, whereas GPX4 protein abundance was higher in term placentas (Figure 1F). Together with the higher iron levels observed in first‐trimester villi, this expression profile is consistent with greater ferroptosis‐associated redox sensitivity during early placental development.

### 3.2 Gestational Changes in Iron Homeostasis and Ferroptosis‐Associated Signaling in the Murine Placenta

We next characterized the spatial distribution and gestational changes of iron homeostasis in the mouse placenta. Immunofluorescence analysis revealed strong ferritin immunoreactivity within the ectoplacental cone (EPC) at GD8.5 and, at later gestational stages, within the decidua and spongiotrophoblast regions (Figure 2A). Biochemical analysis showed that total iron and ferric iron (Fe^3+^) levels were highest at GD8.5, declined by GD12.5, and subsequently increased toward late gestation (Figure 2B).

**FIGURE 2:**
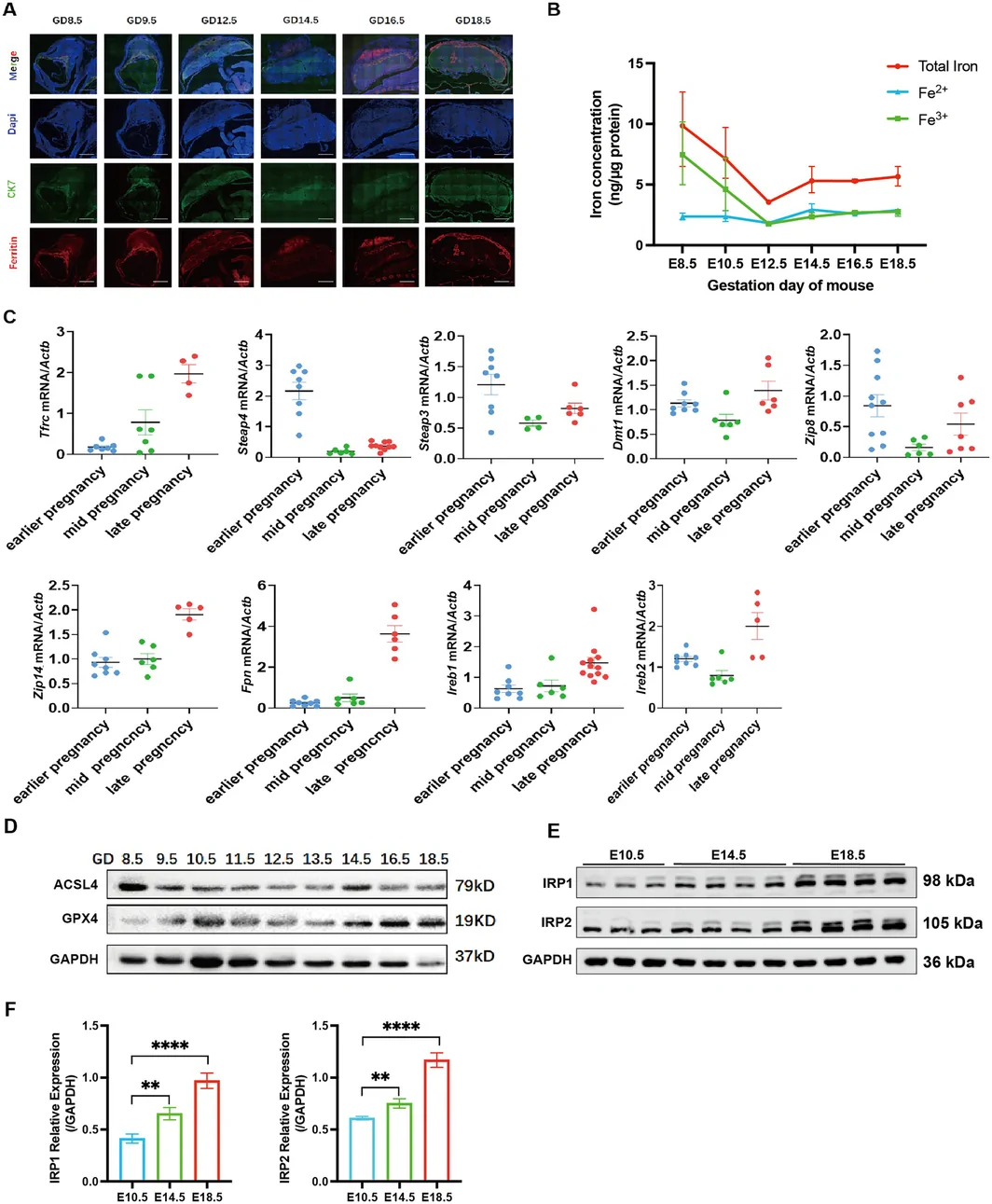
Gestational changes in iron homeostasis and ferroptosis‐associated signaling in the murine placenta. (A) Immunofluorescence staining of ferritin (red), CK7 (green), and DAPI (blue) in mouse placental sections of different gestational stages. EPC, ectoplacental cone; Dec, decidua; Lab, labyrinth; Spo, spongiotrophoblast. (B) Iron concentration in mouse placentas at different gestational stages. Scale bar = 100 μm. (C) The transcription levels of iron uptake and transport‐related genes (*Tfrc, Dmt1, Zip8, Zip14*), the iron exporter gene (*Fpn*), iron reduction‐related genes (*Steap3/4*), and iron regulatory genes (*Ireb1* and *Ireb2*) in mouse placentas at different gestation stages (GD8.5–18.5; *n* = 4) were analyzed by qPCR. (D) Western blot analysis of ACSL4 and GPX4 expression level in mouse placentas at different gestation stages. (E) Western blot of IRP1 and IRP2 protein levels in mouse E10.5, E12.5 and E18.5 placenta. (F) Quantification of IRP1/IRP2 protein levels normalized to GAPDH. Data are presented as mean ± SD (*n* ≥ 3 independent experiments), the *t*‐test, **p < 0.01, ****p < 0.0001.

The mRNA levels of *Tfrc*, which mediates transferrin‐bound iron uptake, increased progressively during gestation. In contrast, expression of the metal‐ion transporter gene *Slc39a8* (*Zip8*) was highest during early gestation and decreased thereafter. The ferrireductase genes *Steap3* and *Steap4* exhibited a similar early gestation‐enriched expression pattern, whereas expression of the iron exporter gene *Slc40a1* (*Fpn*) gradually increased toward late gestation (Figure 2C). Consistent with the human findings, ACSL4 protein levels decreased from GD8.5 to GD18.5, whereas GPX4 expression progressively increased (Figure 2D).

We next assessed the protein abundance of the iron regulatory proteins IRP1 and IRP2 by Western blotting. The results showed that IRP1 and IRP2 protein abundance increased significantly from E10.5 to E18.5 in mouse placentas (Figure 2E,F). To further explore endogenous sources contributing to placental iron regulation, the BACH1/HO‐1 signaling axis was evaluated. BACH1 protein abundance remained relatively stable across gestation, whereas HO‐1 protein expression was highest at E10.5 and gradually declined at later stages (Figure S1A,B). Consistent with protein expression, *Hmox1* and the BACH1‐responsive gene *Nqo1* were most highly expressed at E10.5 and decreased thereafter, while *Fth* expression exhibited a modest increasing trend without reaching statistical significance (Figure S1C).

To directly assess the placental labile iron pool (LIP), Calcein‐AM–based flow cytometric analysis was performed in placentas collected at E12.5, E14.5, and E18.5. Placental LIP levels showed a modest increasing trend during gestation, with the lowest level observed at E12.5 and slightly higher levels at E14.5 and E18.5, generally paralleling total iron measurements, although no statistically significant differences were detected (Figure S1D,E).

### 3.3 Spatially Distinct Expression of Ferroptosis‐Associated Markers During Placentation

To examine the spatial distribution of ferroptosis‐associated markers during placentation, immunofluorescence staining for ACSL4 and GPX4 was performed. In human placental tissues, ACSL4 expression was markedly higher in first‐trimester villi and significantly decreased in term placentas, whereas GPX4 expression remained relatively stable across gestation (Figure 3A,B).

**FIGURE 3:**
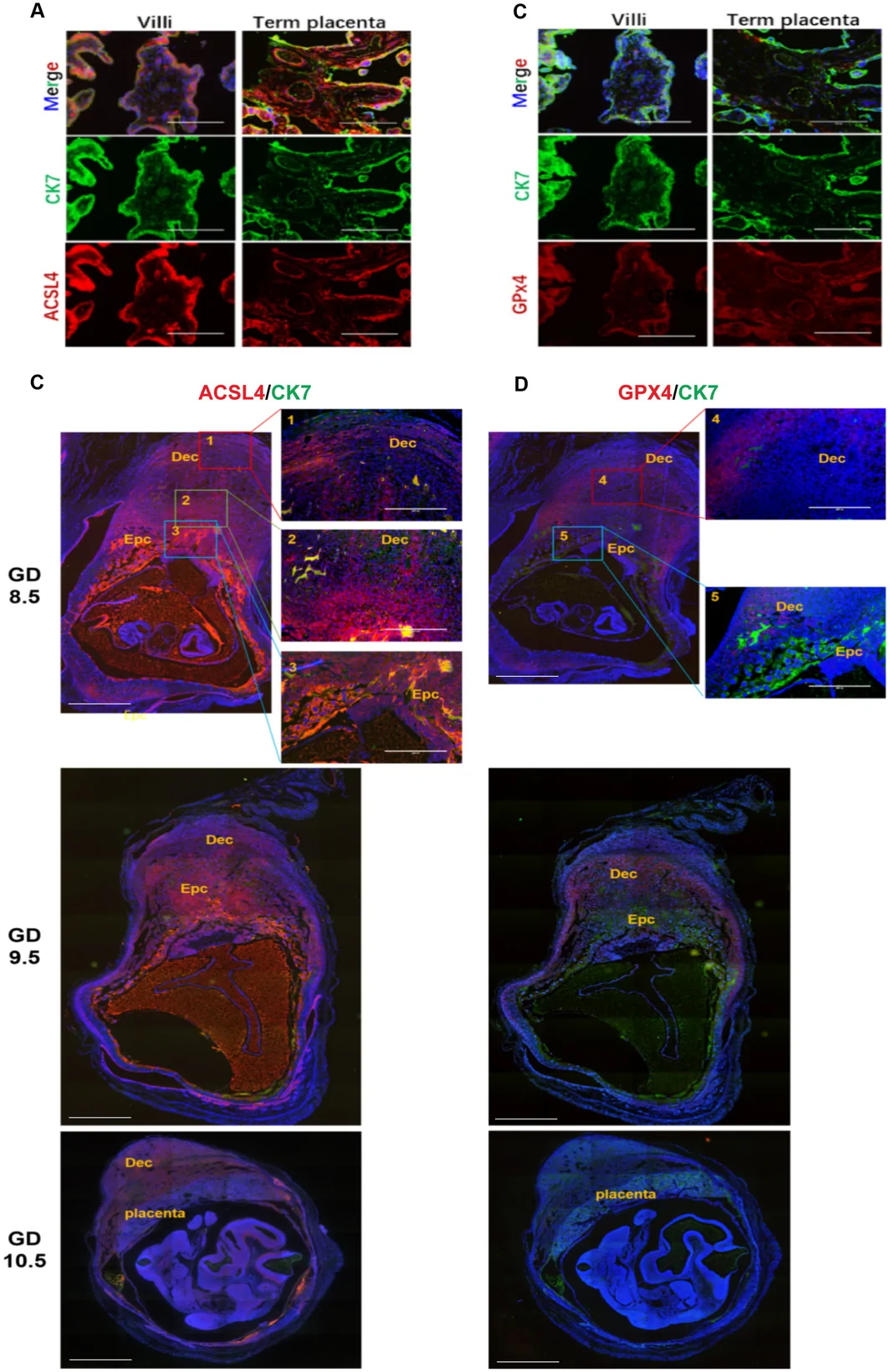
Spatial expression patterns of the ferroptosis‐associated proteins ACSL4 and GPX4 in murine fetoplacental units. (A, B) Immunofluorescence staining of ACSL4 (A, red) or GPX4 (B, red) with CK7 (green) in human villi and term placentas. Scale bar = 100 μm. (C) Immunofluorescence staining of ACSL4 (red) and CK7 (green) in mouse implantation sites at GD8.5, 9.5, and 10.5, scale bars = 1 mm. Higher‐magnification views of the implantation sites at GD8.5 are shown in the box on the right, scale bar = 400 μm. (D) Immunofluorescence staining of GPX4 (red) and CK7 (green) in mouse implantation sites at GD8.5, 9.5, and 10.5, scale bar = 1 mm. Higher‐magnification views of the implantation sites at GD8.5 are shown in the box on the right. Scale bar = 400 μm.

In mouse placentas from GD8.5 to GD10.5, ACSL4 and GPX4 exhibited distinct spatial distribution patterns (Figure 3C and 3D). ACSL4 was predominantly enriched in the vicinity of the ectoplacental cone (EPC) and within putative trophoblast giant cell‐rich regions (Figure 3C). In contrast, GPX4 expression was primarily localized to the outer decidual layer and was relatively sparse in peri‐EPC regions (Figure 3D). These findings are consistent with the spatially distinct distribution of ferroptosis‐associated markers during placentation.

### 3.4 Mild Ferroptosis‐Associated Redox Perturbation Promotes Trophoblast Migration and Invasion

Given that placental development depends on coordinated trophoblast behaviors, including proliferation, apoptosis, migration, and invasion, we next evaluated the functional effects of ferroptosis‐associated redox perturbation using the HTR‐8/SVneo trophoblast cell line. Treatment with erastin induced significant cell death and suppressed proliferation only at higher concentrations (10 and 100 μM) (Figure 4A,B). By contrast, low‐dose erastin (0.1 μM) significantly increased intracellular lipid peroxidation and reactive oxygen species (ROS) production without overt cytotoxicity (Figure 4C,D).

**FIGURE 4:**
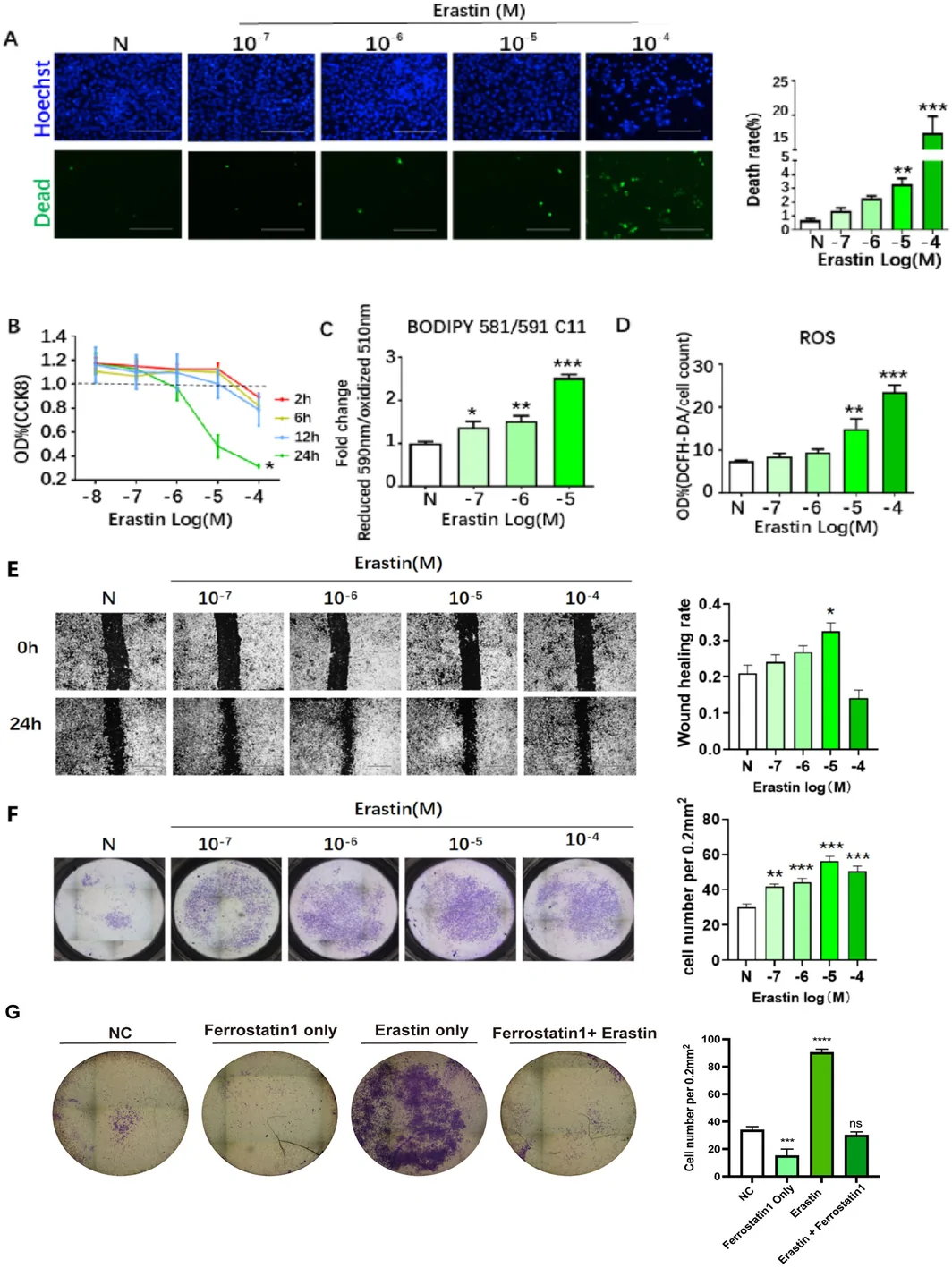
Low‐dose erastin promotes pro‐invasive trophoblast behavior through ferroptosis‐associated redox perturbation HTR‐8/SVneo cells were treated with erastin for 24 h. (A) Cell viability (Live/Dead assay; green = dead cells). (B) Cell proliferation, CCK‐8 assay. HTR‐8/SVneo cells treated with different concentrations of erastin for 2, 6, 12 and 24 h were measured. (C) Lipid peroxidation (BODIPY C11 assay). (D) ROS levels (DCFH‐DA staining). (E) Cell migration (wound healing assay). (F) Cell invasion (Transwell assay). Data are presented as mean + SD (*n* = 3–4 per group), One‐way ANOVA, *p < 0.05, **p < 0.01, ***p < 0.001. (G) Cell invasion (Transwell assay). Data are presented as mean + SD (*n* = 3 per group). One‐way ANOVA, ***p < 0.001, ****p < 0.0001.

Importantly, at this sub‐lethal concentration, erastin enhanced trophoblast migration and invasion in a dose‐dependent manner (Figure 4E,F), suggesting that mild ferroptosis‐associated redox perturbation may promote pro‐invasive trophoblast behavior. To further evaluate whether these effects were linked to ferroptosis‐associated lipid peroxidation, HTR‐8/SVneo cells were co‐treated with 0.1 μM erastin and the ferroptosis inhibitor Ferrostatin‐1 (1 μM). Ferrostatin‐1 significantly attenuated the enhanced invasion induced by both low‐dose erastin (Figure 4G) and Fe^2+^ treatment (Figure S2G), supporting the involvement of ferroptosis‐associated redox signaling in regulating trophoblast invasive capacity.

To further assess the physiological relevance of these findings, trophoblast invasion assays were performed under 2% O_2_ conditions to mimic the low‐oxygen environment of early placentation. Consistent with observations under normoxia, low‐dose erastin or Fe^2+^ treatment continued to promote trophoblast invasion under physiological hypoxia (Figure S4).

### 3.5 Iron Chelation Attenuates Ferroptosis‐Associated Trophoblast Invasion

To further evaluate the iron dependence of ferroptosis‐associated redox signaling in trophoblast behavior, ferrous iron (Fe^2+^) was used as an alternative inducer of ferroptosis‐associated redox perturbation. Fe^2+^ treatment increased cell death, lipid peroxidation, and intracellular ROS levels in a dose‐dependent manner (Figure S2A–D). Importantly, at a sublethal concentration (10 μM), Fe^2+^ significantly enhanced trophoblast migration and invasion (Figure S2F,G).

To further assess whether these effects were iron dependent, cells were co‐treated with the iron chelator deferoxamine mesylate (DFOM). DFOM markedly reduced Fe^2+^‐induced cell death and substantially attenuated the enhancement of trophoblast migration and invasion (Figure S3A–D), supporting the involvement of iron‐dependent ferroptosis‐associated redox signaling in regulating trophoblast behavior.

### 3.6 Antioxidant Treatment Attenuates Pro‐Invasive Effects of Ferroptosis‐Associated Redox Perturbation

The mitochondria‐targeted antioxidant MitoQ (0.1 μM) significantly attenuated erastin‐induced cell death and intracellular ROS accumulation (Figure 5A,B). Furthermore, co‐treatment with MitoQ reversed the stimulatory effects of low‐dose erastin on trophoblast proliferation, migration, and invasion (Figure 5C–F), supporting the contribution of ferroptosis‐associated redox signaling and oxidative lipid stress to the regulation of trophoblast behavior.

**FIGURE 5:**
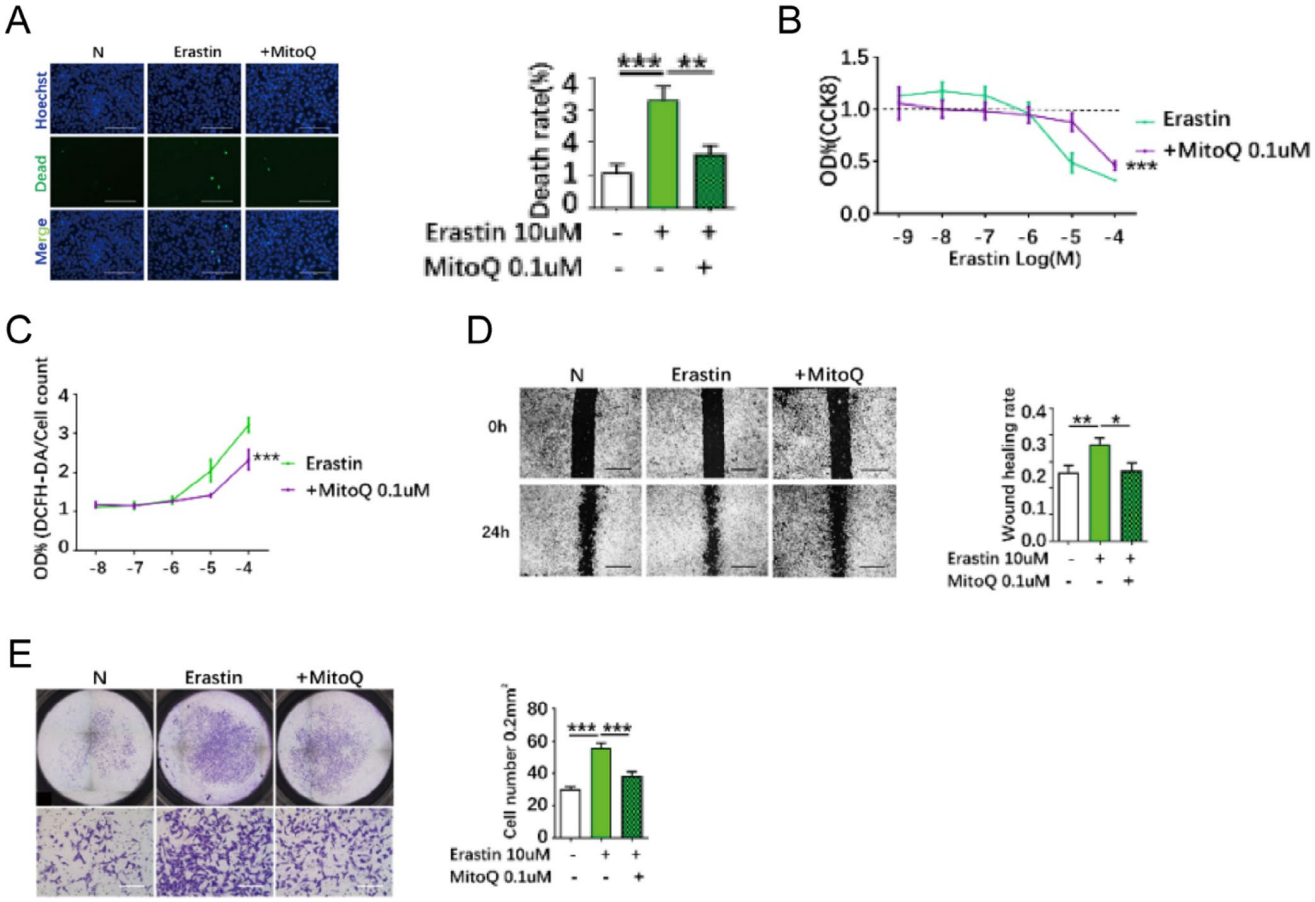
MitoQ attenuates pro‐invasive trophoblast responses induced by ferroptosis‐associated redox perturbation HTR‐8/SVneo cells were treated with 10 μM erastin or combined with 0.1 μM MitoQ. (A) Cell death (Live/Dead staining; green = dead cells). (B) Cell proliferation, CCK‐8 assay. HTR‐8/SVneo cells treated with different concentrations of erastin (combine with or without 10 μM MitoQ) for 24 h were measured. (C) ROS levels (DCFH‐DA staining). (D) Cell migration (wound healing assay). (E) Cell invasion (Transwell assay). Data are presented as mean + SD (*n* = 3–4 per group). One‐way ANOVA or *t*‐test, *p < 0.05, **p < 0.01, ***p < 0.001.

### 3.7 FTH1 and TFRC Knockdown Impairs Trophoblast Responses to Ferroptosis‐Associated Redox Perturbation

To further evaluate the role of iron uptake and iron storage in ferroptosis‐associated trophoblast behavior, the iron storage gene *FTH1* and the iron transporter gene *TFRC* expression were knocked down in HTR‐8/SVneo cells using siRNA. *FTH1* knockdown reduced basal trophoblast proliferation and intracellular ROS levels, whereas *FTH1* and *TRFC* knockdown modestly impaired migration and invasion capacities (Figure 6A–F). Furthermore, subsequent treatment with ferrous iron failed to restore these invasion deficits in *FTH1* or *TFRC*‐deficient cells, supporting the importance of *TFRC*‐mediated iron uptake and *FTH1*‐mediated iron storage in modulating trophoblast responsiveness to ferroptosis‐associated redox signaling.

**FIGURE 6:**
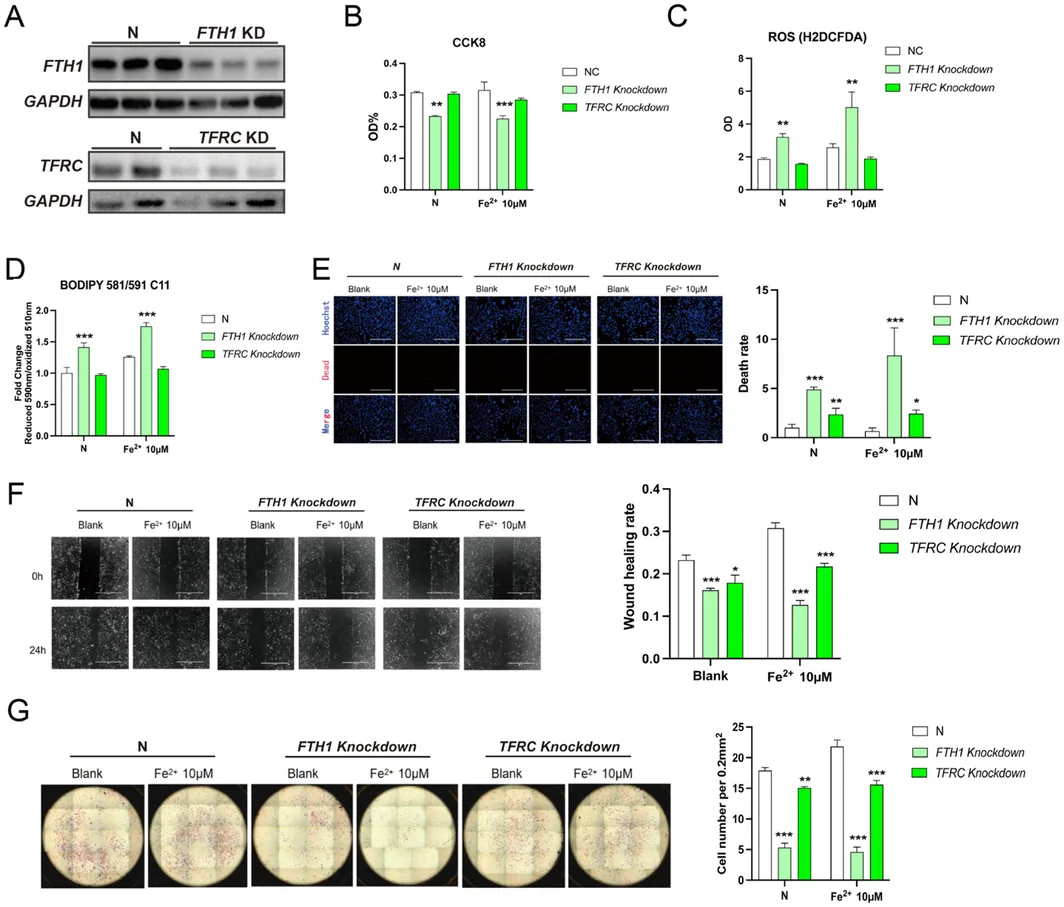
FTH1 and TFRC knockdown impair trophoblast responsiveness to ferroptosis‐associated redox perturbation After knocking down the iron storage gene *FTH1* and the iron transporter gene *TFRC* with siRNA in HTR‐8/SVneo cells, we assess the cellular function. (A) siRNA efficiency was tested by western blot. (B) Cell proliferation, CCK‐8 assay. *FTH1* or *TFRC* knockdown HTR‐8/SVneo cells treat with or without 10 μM Fe^2+^, then tested for CCK‐8. (C) ROS levels (DCFH‐DA staining). (D) Lipid peroxidation (BODIPY C11 assay). (E) Cell death (Live/Dead staining; green = dead cells). (F) Cell migration (wound healing assay). (G) Cell invasion (Transwell assay). Data are presented as mean + SD (*n* = 3–4 per group). One‐way ANOVA or *t*‐test; *p < 0.05, **p < 0.01, ***p < 0.001.

## 4 Discussion

Human and rodent placentas share substantial similarities in structural organization and physiological function, making murine models highly informative for investigating placental development and iron metabolism [8]. Consistent with the conserved regulation of iron homeostasis between species [2], our analyses revealed a coordinated placental program favoring iron availability during early gestation. In human first‐trimester villi, the higher transcription levels of several iron uptake‐ and transport‐related genes, together with greater ferritin abundance and a lower *FPN* transcription level than in term placentas, were consistent with an iron‐handling profile favoring iron acquisition and storage during early placental development. In mouse placentas, however, the gestational patterns were more complex: the transcription level of *Zip8* and the ferrireductase genes *Steap3* and *Steap4* was higher during early gestation, whereas the level of *Tfrc*, *Dmt1*, and *Fpn* increased toward later gestation. These species‐ and gestational stage‐dependent expression patterns were accompanied by dynamic changes in placental iron levels, suggesting that distinct iron‐handling mechanisms operate across placental development [23, 24, 25].

Furthermore, at the protein level, neither IRP1 nor IRP2 differed significantly between first‐trimester villi and term placentas in humans, while in mouse placentas, IRP1 and IRP2 protein abundance increased with advancing gestation. IRP1 and IRP2, as iron regulatory proteins, regulate cellular iron homeostasis through their binding to iron‐responsive elements (IREs) in target mRNAs post‐transcriptionally, so the IRPs' activity cannot be inferred solely from *IREB1/2 (Ireb1/2)* transcript levels or total IRP1/2 protein abundance. We also observed a species‐related difference in IRP protein abundance. This discrepancy may reflect species‐specific regulation, differences in placental structure and developmental timing, or differences in the gestational stages represented by the human and mouse samples. Accordingly, the mouse findings should not be directly extrapolated to human placental IRP regulation. Moreover, because IRP RNA‐binding activity was not directly assessed, the functional significance of these species‐specific expression patterns remains to be determined.

Beyond transporter‐mediated iron uptake, endogenous heme degradation may represent an additional source of placental iron. In mouse placenta, HO‐1, the rate‐limiting enzyme in heme degradation that can release intracellular Fe^2+^, showed its highest protein abundance during early gestation and declined thereafter. This pattern was accompanied by higher early‐gestation expression of *Hmox1* and the oxidative stress‐responsive gene *Nqo1* in mouse placenta. These findings suggest a potential association between heme metabolism and gestational changes in placental iron homeostasis [23, 24, 25]. However, because heme degradation and heme‐derived iron release were not directly measured, the present data do not establish a causal contribution of the HO‐1 pathway to placental iron levels.

Interestingly, despite gestational changes in placental iron abundance, iron transport‐related gene expression, and HO‐1 protein abundance, placental labile iron pool (LIP) levels showed only modest, statistically nonsignificant variation across the examined gestational stages. These findings are consistent with the maintenance of redox‐active iron within a relatively stable range, although they do not directly establish an active buffering mechanism. Such stability may help balance the iron requirements of placental development against the risk of excessive iron‐dependent oxidative stress. The biological effects of placental iron homeostasis may therefore depend not only on the absolute size of the LIP but also on cellular sensitivity to relatively subtle iron‐dependent redox perturbations.

The physiological implications of placental iron regulation are potentially twofold. First, iron serves as an essential cofactor for enzymes involved in cellular metabolism, mitochondrial function, and energy production, all of which are indispensable for highly proliferative and invasive trophoblast populations. Second, even subtle changes in redox‐active ferrous iron may influence cellular redox signaling by facilitating Fenton chemistry, thereby contributing to reactive oxygen species (ROS) generation and lipid peroxidation. Rather than necessarily reflecting overt ferroptotic cell death, such biochemical conditions may increase trophoblast susceptibility to ferroptosis‐associated redox signaling.

The spatial distribution of these key regulators within murine fetoplacental units provided additional support for spatially distinct ferroptosis‐associated redox regulation. ACSL4 was predominantly localized to the invasive trophoblast lineage, particularly trophoblast cells surrounding the ectoplacental cone, regions characterized by active cellular remodeling and invasion. In contrast, GPX4 expression was relatively limited in these regions but enriched in the surrounding decidual compartment.

The spatial distribution of ACSL4 and GPX4 within murine fetoplacental units revealed regional heterogeneity in the expression of ferroptosis‐associated markers. ACSL4 was enriched in invasive trophoblast populations, particularly trophoblast cells surrounding the ectoplacental cone, a regions characterized by active tissue remodeling and invasion, whereas GPX4 expression was comparatively enriched in the surrounding decidual compartment. This spatial separation suggests that trophoblast and decidual compartments may differ in ferroptosis‐associated redox susceptibility. Specifically, enrichment of ACSL4 within invasive trophoblast populations may reflect increased sensitivity to iron‐dependent lipid remodeling and redox signaling, whereas higher GPX4 expression in maternal decidual tissues may contribute to limiting excessive lipid peroxidation and preserving tissue homeostasis. Rather than demonstrating ferroptotic activity, these spatial expression patterns suggest regional differences in ferroptosis‐associated molecular features that may influence trophoblast responses to iron‐dependent redox perturbation during placental development.

Consistent with this interpretation, trophoblast functions remained responsive to mild ferroptosis‐associated perturbation in vitro despite limited gestational differences in placental LIP. Low concentrations of erastin or ferrous iron enhanced trophoblast migration and invasion without overt cytotoxicity, suggesting that mild ferroptosis‐associated redox stress may act as a permissive signal for trophoblast behavior rather than inducing cell death. Importantly, these pro‐invasive effects were attenuated by ferrostatin‐1, the iron chelator deferoxamine mesylate (DFOM), and the mitochondria‐targeted antioxidant MitoQ, supporting the contribution of lipid peroxidation, iron availability, and redox signaling to trophoblast functional regulation. Furthermore, *FTH1* or *TFRC* knockdown blunted trophoblast responsiveness to iron, highlighting the importance of transporter‐mediated iron uptake and storage in modulating ferroptosis‐associated trophoblast behavior.

Importantly, the pro‐invasive effects of mild ferroptosis‐associated redox perturbation were preserved under physiologically relevant hypoxic conditions. Given that early placentation occurs within a low‐oxygen microenvironment, we further evaluated trophoblast invasion under 2% O_2_ conditions and observed effects consistent with those under normoxia. These findings strengthen the physiological relevance of our in vitro observations and suggest that trophoblast behavior remains responsive to subtle iron‐dependent redox signaling within the oxygen environment characteristic of early placentation.

The concept that ferroptosis‐associated redox signaling may exert context‐dependent physiological functions is increasingly appreciated [26]. Similar to autophagy, whose biological consequences range from adaptive homeostatic responses to cell death depending on cellular context and magnitude of the stimulus [27], our findings suggest that sublethal iron‐dependent redox perturbation may modulate trophoblast behavior without inducing overt ferroptotic cell death. Specifically, moderate iron‐dependent lipid peroxidation may influence trophoblast proliferation, migration, and invasion, although its physiological relevance during placental development remains to be established.

In this context, both insufficient and excessive ferroptosis‐associated redox activity may theoretically be detrimental. Reduced iron availability or excessive suppression of physiological redox signaling could impair trophoblast adaptation and invasion, whereas excessive or dysregulated ferroptotic stress may contribute to pathological placental remodeling. Although speculative, these possibilities may have relevance to disorders associated with abnormal placentation, including preeclampsia (PE), fetal growth restriction (FGR), and placenta accreta spectrum [28, 29].

Our findings also raise the possibility that iron availability and antioxidant balance during early gestation may influence placental development in a stage‐dependent manner. However, given the exploratory nature of the present study and the absence of direct in vivo causal evidence, these findings should not be interpreted as evidence against clinically indicated iron supplementation or antioxidant use. Rather, they underscore the need for a more nuanced understanding of the context‐dependent roles of iron and redox signaling during early placentation.

Future studies should directly manipulate ferroptosis‐associated redox signaling in vivo through genetic or pharmacological approaches to determine whether modulation of ferroptosis regulators influences placental development and pregnancy outcomes. Furthermore, elucidating how iron‐dependent lipid peroxidation interfaces with transcriptional and signaling programs governing trophoblast invasion, proliferation, differentiation, and syncytialization will be an important next step.

## Author Contributions

Wenxin Jiang, Yike Yang, and Xiao Yuan conceived and designed the research. Wei Deng, Mark D. Kilby, and Philip N. Baker performed the research and acquired the data. Chao Tong and Yi Yang analyzed and interpreted the data. All authors were involved in drafting and revising the manuscript.

## Funding

This work was supported by NSFC, Excellent Young Scientists Fund (Young Scientists Fund), 82401971 and the National Natural Science Foundation of China (NSFC), U23A20406.

## Disclosure

The authors have nothing to report.

## Conflicts of Interest

The authors declare no conflicts of interest.

## Supporting information


**Figure S1:** Gestational changes in placental HO‐1 and BACH1 protein abundance and the labile iron pool. (A) Western blot of BACH1 and HO‐1 protein levels in mouse E10.5, E12.5, and E18.5 placenta. (B) Quantification of BACH1 and HO‐1 protein levels normalized to GAPDH. Data are presented as mean ± SD (*n* ≥ 3 independent experiments). The *t*‐test, *p < 0.05, **p < 0.01. (C) qPCR analysis of *Hmox1*, *Nqo1*, and *Fth* transcription levels in mouse placental tissues of E10.5, E14.5, and E18.5 (*n* ≥ 3). (D) Representative flow cytometry plots of Calcein‐AM fluorescence intensity in mouse placental cells at E12.5, E14.5, and E18.5. (E) Quantification of intracellular labile iron pool (LIP) in E12.5, E14.5, and E18.5 mouse placental cells using Calcein‐AM and flow cytometry. DFO‐mediated recovery of Calcein‐AM fluorescence intensity was used to estimate intracellular LIP levels. Data are presented as mean ± SD (*n* ≥ 3).
**Figure S2:** Low‐dose ferrous iron promotes pro‐invasive trophoblast behavior through ferroptosis‐associated redox perturbation. HTR‐8/SVneo cells treated with different concentrations of Fe^2+^ for 24 h. (A) Cell viability (Live/Dead assay; green = dead cells). (B) Cell proliferation (CCK‐8 assay). (C) Lipid peroxidation (BODIPY C11 assay). (D) ROS levels (DCFH‐DA staining). (E) Analysis of apoptosis/necrosis rate by flow cytometry. (F) Cell migration (wound healing assay). (G) Cell invasion (Transwell assay). Data are presented as mean + SD (*n* = 3–4). One‐way ANOVA, *p < 0.05, **p < 0.01, ***p < 0.001. (H) HTR‐8/SVneo cells co‐treated with Fe^2+^ and Ferrostatin‐1. Cell invasion (Transwell assay). Data are presented as mean + SD (*n* = 3). One‐way ANOVA, ****p < 0.0001.
**Figure S3:** DFOM attenuates iron‐induced pro‐invasive trophoblast behavior. (A) Viability of cells treated with 10 μM Fe^2^⁺ ±1 μM DFOM (Live/Dead assay; green = dead cells). (B) Cell proliferation (CCK‐8 assay). (C) Cell migration (wound healing assay). (D) Cell invasion (Transwell assay). Data are presented as mean + SD (*n* = 3–4). Two‐way ANOVA or *t*‐test; *p < 0.05, **p < 0.01, ***p < 0.001.
**Figure S4:** Pro‐invasive effects of Erastin and ferrous iron are preserved under physiological hypoxia. (A) Representative images and quantification of Transwell invasion assays in HTR‐8/SVneo cells treated with different concentrations of Erastin. (B) Representative images and quantification of Transwell invasion assays in HTR‐8/SVneo cells treated with different concentrations of Fe^2+^. Data are presented as mean + SD (*n* = 3 independent experiments). One‐way ANOVA was used for statistical analysis. *p < 0.05, **p < 0.0001.

## Data Availability

Privacy/ethical restrictions.
