# Supplementary material for: Gestational Changes in Placental Iron Homeostasis are Associated With Ferroptosis‐Associated Redox Signaling and Trophoblast Behavior
Source: FASEB J. 2026 Sep 28;40(19):e72310. doi: 10.1096/fj.202504881RR (PMC13618451; doi:10.1096/fj.202504881RR)
Supplement: Supplementary file 1 [file FSB2-40-e72310-s001.zip › fsb272310-sup-0001_FigureS1-S4/fsb272310-sup-0005_FiguresS1-S4.docx]

**Supplementary Figure 1. Gestational changes in placental HO-1 and Bach1 protein abundance and the labile iron pool.**

**A.** Western bolt of BACH1 and HO-1 protein levels in mouse E10.5, E12.5 and E18.5 placenta. **B.** Quantification of BACH1 and HO-1 protein levels normalized to GAPDH. Data are presented as mean ± SD (n >= 3 independent experiments). T-test, *p < 0.05, **p < 0.01. **C.** qPCR analysis of *Hmox1*, *Nqo1*, and *Fth* transcription levels in mouse placental tissues of E10.5, E14.5, and E18.5 (n >=3). **D.** Representative flow cytometry plots of Calcein-AM fluorescence intensity in mouse placental cells at E12.5, E14.5, and E18.5. **E.** Quantification of intracellular labile iron pool (LIP) in E12.5, E14.5, and E18.5 mouse placental cells using Calcein-AM and flow cytometry. DFO-mediated recovery of Calcein-AM fluorescence intensity was used to estimate intracellular LIP levels. Data are presented as mean ± SD (n >= 3).

**Supplementary Figure 2. Low-dose ferrous iron promotes pro-invasive trophoblast behavior through ferroptosis-associated redox perturbation**

HTR8/SVneo cells treated with different concentration of Fe²⁺ for 24 hours. **A.** Cell viability (Live/Dead assay; green = dead cells). **B**. Cell proliferation (CCK8 Assay). **C.** Lipid peroxidation (BODIPY C11 assay). **D**. ROS levels (DCFH-DA staining). **E.** Analysis apoptosis/necrosis rate by flow cytometry. **F**. Cell migration (wound healing assay). **G**. Cell invasion (Transwell assay). Data are presented as mean + SD (n = 3–4). One-way ANOVA, *p < 0.05, **p < 0.01, ***p < 0.001. **H**. HTR8/SVneo cells co-treated with Fe^2+^ and Ferrostatin1. Cell invasion (Transwell assay). Data are presented as mean + SD (n = 3). One-way ANOVA, ****p < 0.0001.

**Supplementary Figure 3. DFOM attenuates iron-induced pro-invasive trophoblast behavior**

**A.**Viability of cells treated with 10μM Fe²⁺ ± 1 μM DFOM (Live/Dead assay; green = dead cells). **B**. Cell proliferation (CCK8 Assay). **C**. Cell migration (wound healing assay). **D**. Cell invasion (Transwell assay). Data are presented as mean + SD (n = 3–4). Two-way ANOVA or t-test; *p < 0.05, **p < 0.01, ***p < 0.001.

**Supplementary Figure 4. Pro-invasive effects of Erastin and ferrous iron are preserved under physiological hypoxia**

**A**. Representative images and quantification of Transwell invasion assays in HTR8-S/Vneo cells treated with different concentrations of Erastin. **B**. Representative images and quantification of Transwell invasion assays in HTR8-S/Vneo cells treated with different concentrations of Fe²⁺. Data are presented as mean + SD (n = 3 independent experiments). One-way ANOVA was used for statistical analysis. *p < 0.05, ****p < 0.0001.**
